# Spontaneous ileocecal perforation induced by deep endometriosis

**DOI:** 10.5935/1518-0557.20180087

**Published:** 2019

**Authors:** Sedighe Hosseini, Reza Asemi, Fakhrolmolouk Yassaee, Parya Bamany Moghaddam

**Affiliations:** 1 Preventative Gynecology Research Center (PGRC), Shahid Beheshti University of Medical Sciences, Tehran, Iran; 2 Shahid Beheshti University of Medical Sciences, Tehran, Iran

**Keywords:** Intestinal, endometriosis, bowel, abdominal pain

## Abstract

Bowel endometriosis is a rare condition that may cause catastrophic complications necessitating immediate medical attention. This report describes the case of a patient diagnosed with endometriosis-induced bowel perforation. Albeit rare, bowel perforations caused by endometriosis should be considered in the differential diagnosis of women of reproductive age with abdominal pain.

## INTRODUCTION

Endometriosis is the proliferation of endometrial glands in different parts of the peritoneum, causing inflammation, scars, infertility, and pain (Donnez *et al*., 2002). The condition affects 2-10% of women of reproductive age (Giudice & Kao, 2004). Symptoms may vary from one individual to the next, but they appear mostly during the menstrual period. They range from dysmenorrhea, chronic pelvic pain, infertility, and dyspareunia to bowel disturbances. The rectum and the sigmoid colon are often involved, while the ileum is rarely compromised (4.1%) (Tong *et al*., 2013). Most patients with endometriosis are diagnosed between the ages of 34 and 40 years (Dimoulios *et al*., 2003). The diagnosis of bowel endometriosis is not straightforward, since there is no specific sign to look for in preoperative examination (Dimoulios *et al*., 2003). The patient featured in this case had deep endometriosis of the terminal ileum with perforation and peritonitis. Although most endometriosis-induced bowel perforations occur in the puerperal period, our patient was not pregnant.

##  CASE REPORT

A 47-year old woman was admitted in our emergency department after suffering from severe abdominal pain in the hypogastric and pelvic regions for two weeks. Her condition worsened significantly three days prior to admission. She was vomiting and had nausea and anorexia. Her last period had occurred three weeks prior to admission. The patient was pale and feeling ill, but did not show signs of toxicity. Her heart rate was consistently at 110; her blood pressure was 100/70 mmHg; her oral temperature was 38ºC; and her tilt test was negative. Her abdomen was soft, non-distended, and she complained of mild tenderness to palpation. On auscultation, her bowel sounds were normal. Rectal and vaginal examinations were unremarkable. Chest X rays and ECG were both normal. Baseline workup revealed a WBC of 9700, Hb of 11, and a platelet count of 45300. Amylase, lipase, and aminotransferase readings were normal and the patient had normal bowel movements. She reported a history of dysmenorrhea and dyspareunia started in her teen years. The rest of her medical history was unremarkable and she also had a normal pregnancy 14 years prior. CT scans from 10 days prior to admission showed the large loops of her small bowel were dilated down to the right side of the pelvic cavity. Colonoscopy, endoscopy, and upper gastrointestinal series with Gastrografin carried out a week before hospitalization were normal. Ultrasound examination showed an isoechoic hemorrhagic or endometrial cyst measuring 25×15mm in diameter consistent with a ruptured ovarian cyst, with mild to moderate amounts of fluid in the pelvis and abdomen. The patient was in observation for six hours and her condition deteriorated. Abdominal tenderness worsened, the patient became oliguric, and her Hb level dropped to 9.6.

The tentative diagnosis was acute abdomen due to a ruptured cyst and persistent bleeding. She was sent to the operating room and had her abdomen opened with a midline incision. Approximately two liters of bloody ascites were found, and a sample was sent for cytology testing. A perforation measuring 20mm in diameter was seen in the paramesenteric portion of the ileocecal part of the colon. The perforation was sealed with omentum and was surrounded with pus and fecal material. The patient underwent a right hemicolectomy and a primary end-to-end anastomosis.

**Figure 1:**
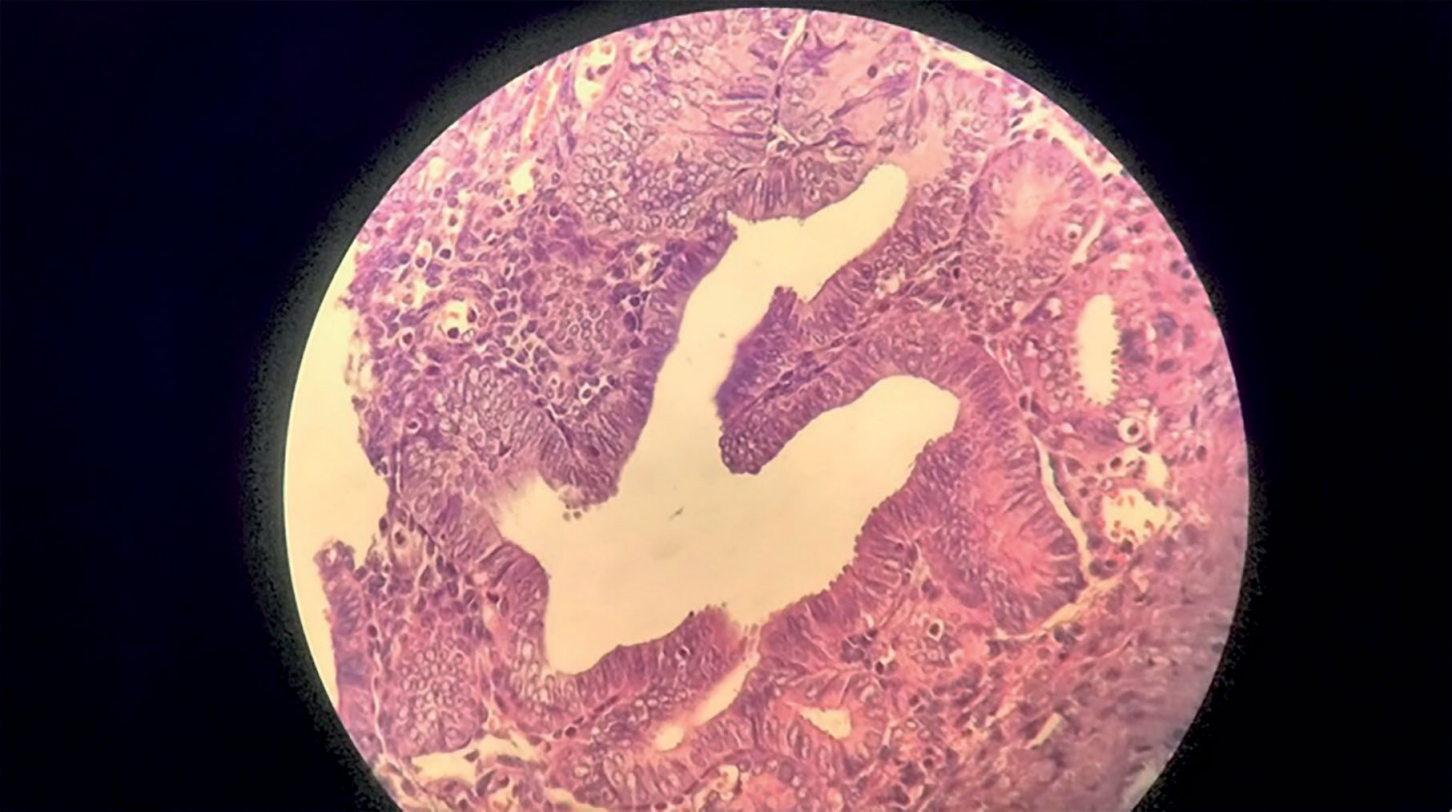
Endometriosis with secondary ulceration, perforation, and inflammatory changes of the bowel wall

Histopathology tests revealed the ascites fluid had blood cells and reactive mesothelial cells and no sign of malignancy. Samples of the terminal ileum, right colon, and omentum revealed endometriosis with secondary ulceration, perforation, and inflammatory changes of the bowel wall (Figure 1). The omentum was unremarkable.

## DISCUSSION

Endometriosis affects 10-15% of women of reproductive age (Tong *et al*., 2013). It is mostly a benign disease, but in rare cases it may cause catastrophic complications needing prompt medical attention. There are three kinds of endometriosis: superficial peritoneal, ovarian (endometrioma), and deeply infiltrating endometriosis.

While the prevalence of endometriosis in asymptomatic patients is unknown, surgical studies of women undergoing unrelated procedures have reported rates ranging between 1 and 7 percent (Sangi-Haghpeykar & Poindexter, 1995). In women with history of endometriosis, rectovaginal or bowel involvement is seen in 5-25% of the cases (Wills *et al*., 2008). In one series the ileum was involved in 2-5% of the individuals with bowel or rectovaginal endometriosis (Bailey *et al*., 1994). According to these authors, the most common symptoms seen in women with bowel endometriosis were abdominal pain, rectal bleeding, palpable or radiographic mass, and dysmenorrhea (Bailey *et al*., 1994). Many women experience diarrhea, constipation, and bloating (Yantiss *et al*., 2001). In rare cases, obstruction (De Ceglie *et al*., 2008), ileus (Bratu *et al*., 2016), intussusceptions (Ranaweera *et al*., 2016), and presumed rectal carcinoma (Rana *et al*., 2016) may occur. Bowel endometriosis primarily affects the serosa and muscle layers of the bowel (Decker *et al.,* 2004), while transmural involvement into the mucosa is rare. Bowel perforation is an uncommon complication that generally occurs during gestation (Pisanu *et al*., 2010). Rising progesterone levels that might decrease implant size can cause perforation in an already inflamed and weakened bowel (Pisanu *et al*., 2010). Transvaginal ultrasound examination may be helpful in diagnosing the condition (sensitivity 43.7%; specificity 50%) (Tong *et al*., 2013). In most cases, it may determine whether the ovaries have been involved. MRI is currently one of the most accurate methods to diagnose bowel endometriosis (sensitivity 77-93%) (Tong *et al*., 2013). Nonetheless, laparoscopy is still the gold standard (Albareda *et al*., 2016).

Surgery is the treatment of choice in complicated bowel endometriosis (obstruction, bleeding, and perforation). The choice of approach is based on surgeon experience and the extension, location, and degree of implant infiltration. Laparoscopy should be attempted whenever possible (Decker *et al*., 2004). Late diagnosis of all forms of endometriosis is still an issue. Future studies should explore better ways to diagnose bowel endometriosis before the rise of complications.

## CONCLUSION

Albeit rare, bowel perforations caused by endometriosis should be considered in the differential diagnosis of women of reproductive age with abdominal pain.
